# Genetic variant in miR‐21 binding sites is associated with colorectal cancer risk

**DOI:** 10.1111/jcmm.14104

**Published:** 2018-12-19

**Authors:** Lisheng Xie, Shuwei Li, Jing Jin, Lei He, Kaili Xu, Lingjun Zhu, Mulong Du, Yanqing Liu, Haiyan Chu, Zhengdong Zhang, Meilin Wang, Danni Shi, Dongying Gu, Min Ni

**Affiliations:** ^1^ Jiangsu Key Lab of Cancer Biomarkers, Prevention and Treatment, Jiangsu Collaborative Innovation Center For Cancer Personalized Medicine Nanjing Medical University Nanjing China; ^2^ The Key Laboratory of Modern Toxicology of Ministry of Education, Department of Genetic Toxicology, School of Public Health Nanjing Medical University Nanjing China; ^3^ Department of Infection Control Zhongshan Hospital Qingpu Branch, Fudan University Shanghai China; ^4^ Department of Cardiovascular Center The Second Affiliated Hospital of Nanjing Medical University Nanjing China; ^5^ Department of Colorectal Surgery The Third Affiliated Hospital of Nanjing University of Chinese Medicine Nanjing China; ^6^ Department of Oncology The First Affiliated Hospital of Nanjing Medical University Nanjing China; ^7^ Department of Biostatistics Nanjing Medical University Nanjing China; ^8^ The Core Facilities School of Public Health Nanjing Medical University Nanjing China; ^9^ Department of Oncology Nanjing First Hospital, Nanjing Medical University Nanjing China

**Keywords:** 3ʹ‐UTR, colorectal cancer, genetic variants, miR‐21

## Abstract

Single nucleotide polymorphisms (SNPs) within binding sites of microRNAs (miRNAs) could modify cancer susceptibility by changing the binding affinity of miRNAs on their target mRNA 3ʹ‐untranslated regions (UTRs). MicroRNA‐21 (miR‐21) is involved in the development of colorectal cancer. However, the relationship between SNPs within the binding sites of miR‐21 and colorectal cancer risk has not been widely investigated. A case‐control study including 1147 patients and 1203 controls was performed to evaluate the association of SNPs in miR‐21 binding sites and colorectal cancer risk. Dual‐luciferase reporter assays and functional assays were performed to evaluate the effects of miR‐21. The SNP rs6504593 C allele conferred an increased risk of colorectal cancer compared with the T allele in an additive model (odds ratio [OR] = 1.19, 95% confidence interval [CI] = 1.04‐1.36, *P* = 0.011). Dual‐luciferase reporter assays demonstrated that the rs6504593 T allele negatively post‐transcriptionally regulated *IGF2BP1* by altering the binding affinity of miR‐21. Additionally, colorectal cancer cells transiently transfected with miR‐21 mimics promoted cell proliferation and suppressed apoptosis, whereas inhibition of miR‐21 decreased cell growth. These data suggest that the miR‐21 binding site SNP rs6504593 in the *IGF2BP1* 3ʹ‐UTR may alter *IGF2BP1* expression and contribute to colorectal cancer risk.

## 1 INTRODUCTION

Colorectal cancer is one of the main causes of cancer‐related mortality in the United States. They are projected to be 140 250 new colorectal cancer cases and 50 630 deaths in 2018.^1^ In China, colorectal cancer is also a public health concern due to the rapid increase in morbidity in recent decades.^2^ Colorectal cancer is known as a complex disease because of environmental and genetic factors and their interactions. Despite enormous efforts that have been devoted to exploring the pathogenesis of colorectal cancer, the precise etiology remains to be elucidated.

MicroRNAs (miRNAs) are endogenous, small non‐coding RNAs (18‐23 nucleotides) that silence gene expression by binding to target mRNA 3ʹ‐untranslated regions (3ʹ‐UTRs).^3^ Previously, a few studies have reported that single nucleotide polymorphisms (SNPs) in miRNA target sites can influence miRNA‐mediated gene regulation and play an important role in tumorigenesis.^4^, ^5^, ^6^ MicroRNA‐21 (miR‐21) was upregulated in various tumors including gastric cancer, colorectal cancer, malignant breast tumor and pancreatic carcinoma.^7^, ^8^, ^9^, ^10^, ^11^ Moreover, miR‐21 affected the processes involved in the progression of colorectal cancer by directly targeting genes including *RHOB*,* PDCD4* and *Cdc25A*.^12^, ^13^, ^14^, ^15^, ^16^

Our previous study found that miR‐21 could be a valuable biomarker for the diagnosis of colorectal cancer,^17^ but the relationship between SNPs within its binding sites and colorectal cancer risk has not been investigated. Therefore, we aimed to investigate whether genetic variants within the binding sites of miR‐21 could alter the susceptibility of patients for colorectal cancer.

## 2 MATERIALS AND METHODS

### 2.1 Study population

We conducted a case‐control study including 1147 colorectal cancer individuals and 1203 cancer‐free controls. In brief, the participants were consecutively recruited from The First Affiliated Hospital and Nanjing First Hospital of Nanjing Medical University beginning in September 2010 and were histopathologically confirmed. The cancer‐free controls were randomly selected from individuals who participated in physical examinations and were frequency‐matched by age (±5 years) and sex. This study protocol was approved by the Institutional Review Board of Nanjing Medical University. Previous studies have described the detailed information of the study subjects.^18^, ^19^

### 2.2 SNPs selection

We focused on both the miR‐21 target genes dataset and 3ʹ‐UTR dataset using the UCSC browser (http://www.genome.ucsc.edu). Using the algorithm TargetScan (http://www.targetscan.org/), we selected 1920 miR‐21 target genes. We further identified 13 SNPs within miR‐21 binding sites using the Chinese Han population in Beijing (CHB) population of the 1000 Genomes Project. The following were three criteria for inclusion: (a) SNPs located in miR‐21 binding sites; (b) minor allelic frequency (MAF) ≥5%; (c) Hardy‐Weinberg equilibrium (HWE) *P* > 0.05. Finally, in accordance with whether miR‐21 target genes were previously reported to be related to cancer risk, five SNPs (rs2273847, rs6504593, rs1049109, rs6108 and rs7337488) were enrolled as candidate SNPs.

### 2.3 SNPs genotyping

Genomic DNA was isolated from venous blood lymphocytes using the Qiagen Blood kit (Qiagen). Custom TaqMan SNP genotyping assays were performed using the 384‐well ABI 7900HT Real‐time PCR System (Applied Biosystems, Foster City, CA, USA). The sequences of primers and probes were designed for each SNP genotyping (Table S1). The SNP analysis was carried out independently by two persons in a blinded fashion. The call rates for all SNPs were over 95%. Additionally, 5% random samples were selected to repeat genotyping for quality control, and the concordant rate for each SNP was 100%.

### 2.4 Plasmid construction and luciferase reporter assays

Two human colorectal cancer cell lines, HCT116 and SW620, were purchased from Shanghai Institute of Biochemistry and Cell Biology, Chinese Academy of Sciences (Shanghai, China). The reporter plasmid containing the sequence of the wild‐type or mutant insulin‐like growth factor 2 messenger RNA binding protein 1 (*IGF2BP1*) 3ʹ‐UTR was constructed and cloned into the *Not*I/*Xh*oI restriction enzyme sites of the psiCHECK‐2 vector (Promega). DNA sequencing was used to confirm all the cloned sequences. For luciferase assays, HCT116 and SW620 cells were seeded in 24‐well plates. After 12 hours, each well of cells was cotransfected with a luciferase vector encompassing the wild‐type or mutant *IGF2BP1* 3ʹ‐UTR fragments and miR‐21 mimics using Lipofectamine 2000 (Invitrogen). At 24 hours after transient transfection, the Dual‐Luciferase Reporter Assay System (Promega) was used to measure the luciferase activity, and the Renilla luciferase activity was normalized to Firefly luciferase activity.

### 2.5 Quantitative reverse transcription polymerase chain reaction

Colorectal tumor tissues and their paired adjacent normal tissues were collected from colorectal cancer subjects. The clinical characteristics are provided in Table S2. Total RNA was extracted from colorectal cancer cells and tissue specimens using TRIzol (Invitrogen) according to the manufacturer's protocol. Reverse transcription was performed with Primescript RT Master Mix (TaKaRa) and RNA PCR Kit (AMV) (TaKaRa). Real‐time polymerase chain reaction (PCR) assay with SYBR Green Master Mix reagent kit (TaKaRa) was performed using a 7900 Real‐time PCR system (ABI). The relative expression of miR‐21, *ALPP* and *IGF2BP1* normalized to *U6* and *ACTINB* was calculated using the 2^−ΔCt^ method. The primer sequences are listed in Table S1.

### 2.6 The cancer genome atlas and eQTL analysis

We obtained miRNA and mRNA expression data and genotypic data of colorectal samples from the cancer genome atlas (TCGA) database. Expression quantitative trait loci (eQTL) analysis was performed to evaluate the associations between SNP genotypes and mRNA expression levels. Only samples that have miR‐21 rs6504593 genotyping data were included for eQTL analysis.

### 2.7 Cell proliferation, apoptosis and cycle assays

HCT116 and SW620 cells were seeded into six‐well plates and transiently transfected with miR‐21 mimics or inhibitor by Lipofectamine 2000 reagent (Invitrogen). For cell proliferation assay, the cells were plated into 96‐well plates (4000 cells/well). At 12 hour intervals, Cell Counting Kit‐8 (CCK‐8, Dojindo) reagent was added and incubated at 37°C for 2 hours in a humidified incubator. The absorbance values at 450 nm, which represented the number of cells, were measured using an Infinite M200 spectrophotometer (Tecan). For cell apoptosis assays, after 48 hours of transfection, cells were collected and stained with the Annexin V apoptosis detection kit (BD Biosciences) following the manufacturer's instruction manual. The percentage of apoptotic cells was analyzed by flow cytometer (FACScan; BD Biosciences). For cell cycle assays, at 48 hours after transfection, cells were harvested, fixed with 75% ice‐cold ethanol at 4°C overnight, and then stained with propidium iodide. The flow cytometer system was used to count and compare the percentage of the cells in G0/G1, S and G2/M phases. Each experiment was performed in triplicate, and all experiments were performed three times independently.

### 2.8 Statistical analysis

Student's *t* test for continuous variables and Pearson's χ^2^ test for categorical variables were used to analyze the differences in the distribution of demographic and clinical characteristics between colorectal cancer cases and controls. Hardy‐Weinberg equilibrium for the selected SNP allele frequencies was evaluated using a goodness‐of‐fit χ^2^ test in the control group. Multivariate logistic regression analysis with adjustments for age, sex, smoking and drinking status was used to evaluate the association of the selected SNPs with colorectal cancer risk by computing adjusted odds ratios (ORs) and 95% confidence intervals (CIs). All experimental results are shown as the mean ± SD, and the statistical comparisons between groups were tested by *t* test, Mann‐Whitney *U* test and ANOVA. sas 9.4.0 (SAS Institute) was used for all the statistical analyses, and statistical significance was set at *P* < 0.05.

## 3 RESULTS

### 3.1 Demographic and clinical characteristics of subjects

The demographic and clinical characteristics of cases and controls are presented in Table S3. No significant differences were detected between colorectal cancer individuals and cancer‐free controls for age (*P* = 0.751), gender (*P* = 0.116), smoking status (*P* = 0.097) and alcohol drinking status (*P* = 0.113). However, a higher proportion of family history of cancers was observed in colorectal cancer cases than in controls (*P* < 0.001). Of all colorectal cancer cases, the frequencies of low, intermediate and high histological grade were 7.4%, 76.7% and 15.9%, respectively. Additionally, 8.4%, 43.1%, 36.8% and 11.7% of colorectal cancer subjects were in Dukes A, B, C and D, respectively.

### 3.2 Association between selected SNPs and colorectal cancer susceptibility

The baseline characteristics of the selected SNPs are summarized in Table S4. The genotype frequencies of selected SNPs (rs2273847, rs6504593, rs1049109 and rs6108), except rs7337488, were in HWE among the controls (*P* > 0.05). Therefore, we chose these four SNPs for further analysis. By multivariate logistic regression analysis with adjustments for sex, age, drinking and smoking status, SNPs rs6504593 and rs1049109 had a significant association with colorectal cancer susceptibility in the additive model (OR = 1.19, 95% CI = 1.04‐1.36, *P* = 0.011 for rs6504593 in *IGF2BP1* and OR = 1.23, 95% CI = 1.07‐1.41, *P* = 0.004 for rs1049109 in *ALPP*). Similar results were observed in dominant and codominant models (Table 1).

**Table 1:** Association of selected SNPs with the risk of colorectal cancer in four genetic models

SNPs	Additive model		Dominant model		Recessive model		Codominant model^b^			
	OR (95% CI)^a^	*P*	OR (95% CI)^a^	*P*	OR (95% CI)^a^	*P*	het		hom	
							OR (95% CI)^a^	*P*	OR (95% CI)^a^	*P*
rs2273847	1.11 (0.98‐1.25)	0.108	1.09 (0.93‐1.29)	0.279	1.27 (0.97‐1.65)	0.085	1.05 (0.88‐1.25)	0.579	1.29 (0.98‐1.71)	0.070
rs6504593	1.19 (1.04‐1.36)	0.011	1.22 (1.03‐1.44)	0.019	1.34 (0.95‐1.89)	0.093	1.19 (1.00‐1.41)	0.053	1.43 (1.01‐2.03)	0.044
rs1049109	1.23 (1.07‐1.41)	0.004	1.28 (1.09‐1.51)	0.003	1.27 (0.87‐1.85)	0.224	1.27 (1.07‐1.51)	0.007	1.38 (0.94‐2.21)	0.099
rs6108	1.14 (0.98‐1.31)	0.087	1.51 (0.98‐2.32)	0.063	1.12 (0.94‐1.33)	0.207	1.44 (0.92‐2.25)	0.114	1.54 (1.00‐2.38)	0.052

CI, confidence interval; OR, odds ratio; SNP, single nucleotide polymorphism.

a Adjusted for age, sex, smoking and drinking status in logistic regression model.

b het: heterozygote vs major homozygote; hom: minor homozygote vs major homozygote.

### 3.3 Stratification analysis of rs6504593 and rs1049109

We further conducted stratification analysis by demographic characteristics to assess the associations of rs6504593 and rs1049109 with colorectal cancer risk under a dominant model. For rs6504593, subjects carrying the TC/CC genotype had an enhanced susceptibility of colorectal cancer in subgroups of non‐smokers (*P* = 0.010), non‐drinkers (*P* = 0.013) and individuals with a family history of cancers (*P* = 0.003), compared with the TT genotype (Table S5). For rs1049109, we identified a significantly increased risk in subgroups of older individuals (*P* = 0.001), females (*P* = 0.011), non‐smokers (*P* = 0.012), non‐drinkers (*P* = 0.002) and individuals both with family history of cancers (*P* = 0.026) and without family history of cancers (*P* = 0.037) for carriers of the CT/TT genotype comparing with the CC genotype (Table S6).

Furthermore, we evaluated the associations of rs6504593 and rs1049109 with colorectal cancer risk stratified by clinical characteristics. For rs6504593, the carriers of the TC/CC genotype had an increased susceptibility for colon tumors (OR = 1.25, 95% CI = 1.02‐1.53), intermediate or well‐differentiated histological grade colorectal cancer (OR = 1.22, 95% CI = 1.02‐1.45 and OR = 1.39, 95% CI = 1.01‐1.90, respectively) and advanced stage cancer (Dukes C and D) (OR = 1.24, 95% CI = 1.01‐1.52) (Table 2). For rs1049109, we identified a significantly increased risk for colon tumors (OR = 1.25, 95% CI = 1.02‐1.53), intermediate histological grade colorectal cancer (OR = 1.27, 95% CI = 1.06‐1.52) and early stage cancer (Dukes A and B) (OR = 1.38, 95% CI = 1.13‐1.68) for carriers of the CT/TT genotype (Table S7).

**Table 2:** Associations between rs6504593 genotype and clinical characteristics of colorectal cancer

Variables	Genotypes		OR (95% CI)^a^	*P* a
	TT, n (%)	TC/CC, n (%)		
Controls (n = 1203)	735 (61.1)	468 (38.9)	1.00	
Cases (n = 1147)	646 (56.3)	501 (43.7)	1.22 (1.03‐1.44)	0.019
Tumor site				
Colon	312 (55.8)	247 (44.2)	1.25 (1.02‐1.53)	0.034
Rectal	334 (56.8)	254 (43.2)	1.19 (0.97‐1.45)	0.092
Histological grade				
Low	54 (63.5)	31 (36.5)	0.90 (0.57‐1.43)	0.666
Intermediate	496 (56.4)	384 (43.6)	1.22 (1.02‐1.45)	0.031
High	96 (52.7)	86 (47.3)	1.39 (1.01‐1.90)	0.042
Dukes stage				
A + B	336 (56.9)	255 (43.1)	1.20 (0.98‐1.46)	0.081
C + D	310 (55.8)	246 (44.2)	1.24 (1.01‐1.52)	0.039

OR, odds ratio; CI, confidence interval.

a Adjusted for age, sex, smoking and drinking status in logistic regression model.

### 3.4 miR‐21 and its target genes’ expression in colorectal cancer tissues

**Figure 1:**
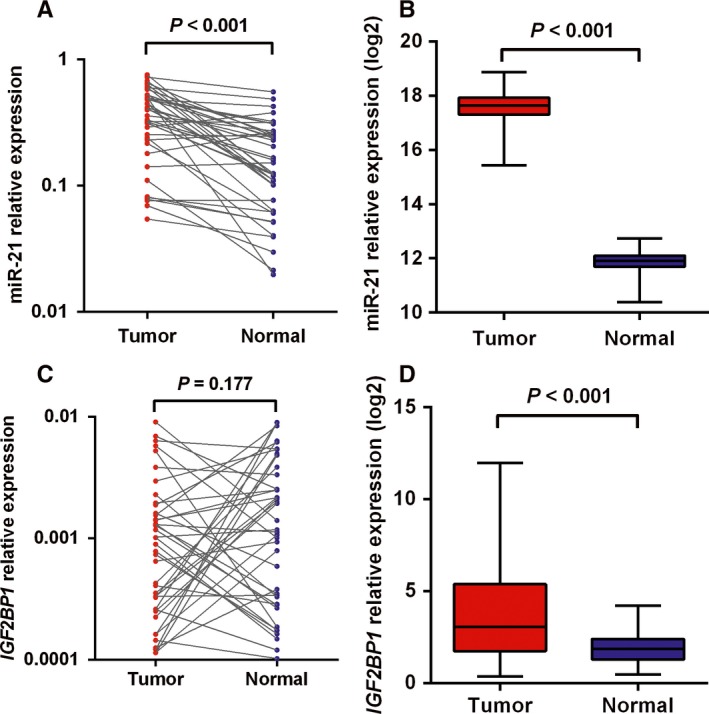
The expression of miR‐21 and *IGF2BP1* in human colorectal cancer tissues. A, The expression of miR‐21 in colorectal cancer tissues and adjacent normal tissues. B, The miR‐21 expression was analyzed from TCGA database. C, The *IGF2BP1* expression levels were evaluated in colorectal cancer tissues and their adjacent normal tissues. D, The *IGF2BP1* expression levels were analyzed from TCGA database. The miR‐21 and *IGF2BP1* expression levels were log2 transformed. The *P* value was calculated using paired *t* test or Mann‐Whitney *U* test

We measured the expression of miR‐21 in colorectal tumor and adjacent normal tissues and found that miR‐21 expression was significantly upregulated in tumor tissues compared to normal tissues (*P* < 0.001) (Figure 1A). This result was consistent with the data obtained from TCGA (*P* < 0.001) (Figure 1B). *IGF2BP1* expression showed no significant difference between colorectal cancer and adjacent normal tissues (*P* = 0.177) (Figure 1C), whereas TCGA data showed that *IGF2BP1* expression in colorectal cancer tissues was significantly higher than that in normal tissues (*P* < 0.001) (Figure 1D). The expression level of *ALPP* in colorectal cancer and adjacent normal tissues was almost undetectable, which was supported by data in TCGA. Therefore, *IGF2BP1* was retained for further analysis. Moreover, a negative association between *IGF2BP1* and miR‐21 expression was discovered in both colorectal tumor tissues (r = −0.348,* P = *0.029) and normal tissues (r = −0.401, *P = *0.010), although no significant correlation was found by using TCGA data (Figure S1).

### 3.5 SNP rs6504593 interfere miR‐21 and *IGF2BP1* 3ʹ‐UTR interaction

**Figure 2:**
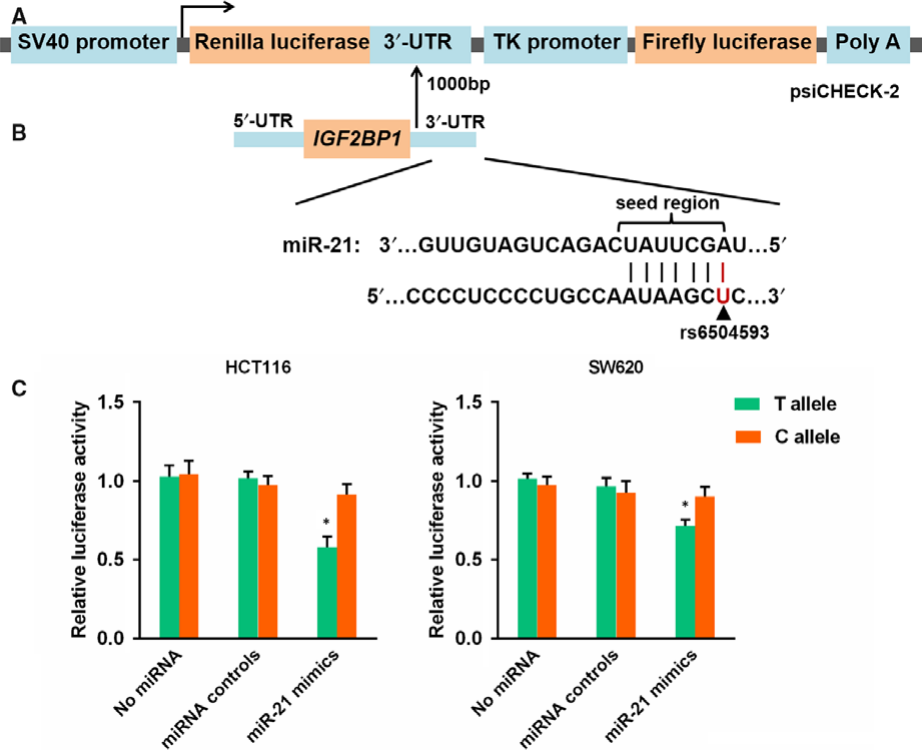
Characterization and functional analysis of the 3ʹ‐UTR of *IGF2BP1*. A, Schematic representation of reporter plasmids containing the *IGF2BP1* 3ʹ‐UTR, which was inserted downstream of the Renilla luciferase gene in the psiCHECK‐2 vector. B, Bioinformatics predicted the binding site between miR‐21 and the *IGF2BP1* 3ʹ‐UTR. C, Luciferase reporter assays was used to measure rs6504593 T or C allele differences with the presence or interference of miR‐21. HCT116 and SW620 cells were transiently cotransfected with constructs and miR‐21 mimics. *, *P* < 0.05.

To demonstrate whether rs6504593 regulates *IGF2BP1* expression by miR‐21, we performed dual‐luciferase reporter assays by constructing psiCHECK‐2 vectors containing wild‐type or mutated‐type *IGF2BP1* 3ʹ‐UTR (Figure 2A). The specific binding sites of *IGF2BP1* 3ʹ‐UTR and miR‐21 were provided by bioinformatics tools (Figure 2B). The results indicated that constructs containing the rs6504593 T allele significantly reduced luciferase activity compared with the C allele in HCT116 and SW620 cells (*P* = 0.003 and *P* = 0.012, respectively) (Figure 2C). These data suggested that miR‐21 may directly target the *IGF2BP1* 3ʹ‐UTR with the rs6504593 T allele.

### 3.6 eQTL analysis of *IGF2BP1*

To understand whether rs6504593 can regulate the expression of *IGF2BP1*, we performed an eQTL analysis in TCGA data. We found that rs6504593 genotypes had no influence on *IGF2BP1* expression in either colorectal cancer tissues or normal tissues (*P* = 0.705 and *P* = 0.774, respectively) (Figure S2).

### 3.7 Functional characteristics of miR‐21 in colorectal cancer cells

**Figure 3:**
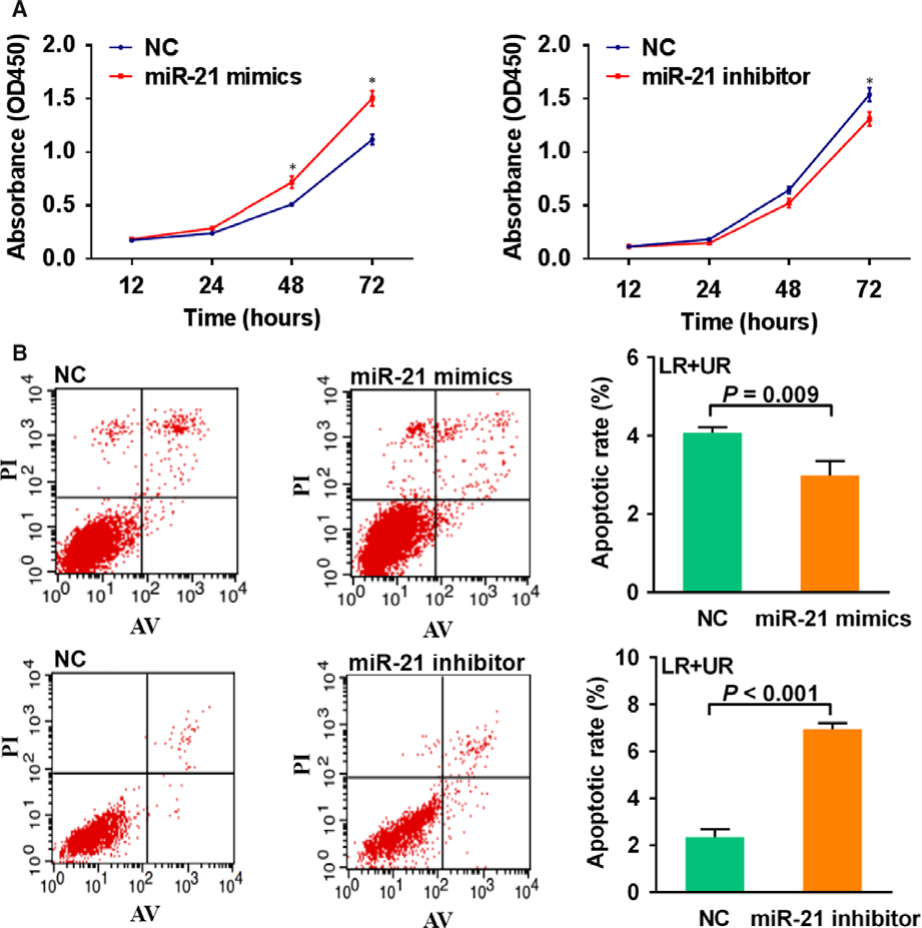
Transient transfection with miR‐21 mimics or inhibitor regulated SW620 cell proliferation and apoptosis. A, Cell proliferation activity was measured by the CCK8 assay. *, *P < *0.05. B, The level of apoptosis was detected by flow cytometry. All bars represent the mean values ± SD. All results are representative of triplicate experiments. The *P* value was calculated by two‐sided *t* test. LR, early apoptotic cells; UR, terminal apoptotic cells.

The functional mechanism of miR‐21 was characterized after transiently transfecting miR‐21 mimics or inhibitor into SW620 and HCT116 cells. SW620 cells transfected with miR‐21 mimics significantly increased cell proliferation (*P* = 0.004) and suppressed cell apoptosis (*P* = 0.009) (Figure 3A,B). SW620 cells transfected with miR‐21 inhibitor significantly decreased cell proliferation (*P* = 0.047) and showed increased cell apoptosis (*P* < 0.001) (Figure 3A,B). Next, HCT116 cells transfected with miR‐21 mimics significantly increased cell proliferation (*P* = 0.012) but did not significantly induce cell apoptosis changes (Figure S3A,B). HCT116 cells transfected with miR‐21 inhibitor significantly decreased cell proliferation (*P* < 0.001) and increased cell apoptosis (*P* = 0.046) (Figure S3A,B). In addition, HCT116 and SW620 cells transfected with miR‐21 mimics did not profoundly alter the cell cycle (Figure S4A,B).

## 4 DISCUSSION

In this study, we investigated the association between genetic variants in miR‐21 binding sites and colorectal cancer susceptibility in a case‐control study. Our finding shows that rs1049109 and rs6504593 were new susceptibility SNPs for colorectal cancer, which have not been reported in previous studies.

IGF2BP1, belonging to the family of RNA‐binding proteins, plays key roles in many biological processes through binding to the key motif in RNA structures.^20^ The abnormal expression of *IGF2BP1* may cause a series of diseases, including malignant tumors.^21^, ^22^ Recent research has reported that *IGF2BP1* expression levels were upregulated in many types of tumors, such as hepatocellular carcinoma, renal cell carcinoma and cutaneous squamous cell carcinoma.^23^, ^24^, ^25^ In our study, the expression of *IGF2BP1* in colorectal cancer tissues, including TCGA data, was almost concordant with previous studies.^23^, ^24^, ^25^ However, no significant difference was detected in our paired colorectal tissues based on a Chinese population. The conflicting data may be caused by the different populations of these two datasets. Moreover, studies have investigated the function of *IGF2BP1* in promoting cell proliferation, apoptosis and invasion in various cancers.^26^, ^27^

Accumulated evidence has shown that genetic variants within binding sites of miRNA could affect susceptibility of various tumors by changing the binding affinity of miRNAs on its target mRNAs 3ʹ‐UTRs.^25^, ^28^ Dual‐luciferase reporter assays indicated that miR‐21 directly targeted the *IGF2BP1* 3ʹ‐UTR in the study. The mutation allele of rs6504593 reduced the binding of miR‐21 to the *IGF2BP1* 3ʹ‐UTR and increased *IGF2BP1* expression, which leads to the initiation of colorectal cancer. MiR‐21 has been reported to be upregulated in various cancers, and it inhibited colorectal cancer cell apoptosis and enhanced cell growth.^12^, ^14^, ^29^, ^30^ Our experimental data were almost identical to previously reported literature. Furthermore, we found a significant negative correlation between miR‐21 and *IGF2BP1* expression, suggesting that miR‐21 could negatively regulate *IGF2BP1*. The relationship between rs6504593 genotypes and relative *IGF2BP1* expression was further analyzed, but we found no significant association. Furthermore, the directed biological function of the combination of miR‐21 and *IGF2BP1* in colorectal cancer should be conformed in the future to identify the functional relationship between miR‐21 and *IGF2BP1*.

After stratification analysis of demographic characteristics, we observed that the rs1049109 CT/TT genotype had an elevated risk among older people, females, non‐smokers, non‐drinkers and individuals both with and without a family history of cancers. Additionally, the rs6504593 TC/CC genotype showed an increased susceptibility among non‐smokers, non‐drinkers and individuals with a family history of cancers. These results indicated that the occurrence of colorectal cancer may be affected by epidemiological factors, environmental exposures and genetic factors. Smoking and drinking are considered risk factors for colorectal cancer,^31^, ^32^, ^33^ but they were observed as protective factors in this study. The relatively small sample size after stratification analysis of smoking status and alcohol consumption may be the reason. Moreover, we conducted stratification analysis by clinical characteristics. The results suggested that the rs6504593 TC/CC genotype had an obviously increased susceptibility in patients with Dukes C/D and that the genetic variants participate in the advanced stage of colorectal cancer. Additionally, the subjects carrying the TC/CC genotype had an increased susceptibility to colorectal cancer in subgroups of colon tumor patients and individuals with an intermediate or well‐differentiated grade. It could be interpreted that different molecular mechanisms resulted in different colorectal cancer grade and site, which caused differences in the susceptibility to colorectal cancer.

## 5 CONCLUSIONS

Here, we have demonstrated that the SNP rs6504593 was associated with colorectal cancer susceptibility. Moreover, the rs6504593 T allele negatively regulated the post‐transcription of *IGF2BP1* by altering the binding affinity of miR‐21. Furthermore, we found that miR‐21 mimics promoted cell proliferation and suppressed apoptosis, whereas an inhibitor of miR‐21 decreased cell growth. Thus, our study provides a novel view of miR‐21‐induced colorectal cancer development. The miR‐21 binding site SNP rs6504593 may be used as a new potential biomarker for the diagnosis of colorectal cancer.

## CONFLICT OF INTEREST

The authors declare no conflicts of interest.

## AUTHOR CONTRIBUTIONS

Dongying Gu and Min Ni conceived and designed the experiments. Lisheng Xie, Shuwei Li and Jing Jin wrote the paper. Lei He, Kaili Xu, Lingjun Zhu, Mulong Du, Zhengdong Zhang and Meilin Wang contributed reagents/materials/analysis tools. Yanqing Liu, Haiyan Chu and Danni Shi recruited samples. All authors reviewed the manuscript.
