# Supplementary material for: Genetic variant in miR‐21 binding sites is associated with colorectal cancer risk
Source: J Cell Mol Med. 2018 Dec 19;23(3):2012–9. doi: 10.1111/jcmm.14104 (PMC6378227; doi:10.1111/jcmm.14104)

**Supplementary Table 1. Primers and probes sequences used in this study**

|  | Description | Sequence (5’-3’) |  |
| --- | --- | --- | --- |
| TaqMan |  | Primer | Probe |
| Genotyping | rs6108 | F: AAATCACTAGCTACCAGCATACAGATG | T allele: FAM-CCATAGCTTAGCCC-MGB |
|  |  | R: GGCCTCAGGGTGGGGAGAT | A allele: HEX-CATAGCATAGCCCCCCT-MGB |
| Genotyping | rs2273847 | F: GGTTAACATCTTACGATTACCCTTTGA | A allele: FAM-TAAGTACAAAGCTTATTCAC-MGB |
|  |  | R: CATTTGTGAAAGTTGTGTTGGATTT | G allele: HEX-AATAAGTACAAAGCTTACTC-MGB |
| Genotyping | rs1049109 | F: GGGGATTTTGACACAGCCTTC | C allele: FAM-CCCACTAAGCTAATTC-MGB |
|  |  | R: ACGTCTGAGAAGTTGAGATTT GGG | T allele: HEX-CCACTAAGTTAATTCCAC-MGB |
| Genotyping | rs6504593 | F: CTATTCCTGCCAGTGAGTCCTTC | C allele: FAM-CCAATAAGCCCCCCC-MGB |
|  |  | R: GAAGTGCCAGTCAAGATTTAAGCAT | T allele: HEX-CCAATAAGCTCCCC-MGB |
| Genotyping | rs7337488 | F: CCTCAGAGTTAATGGTCTTGGTAGTCT | G allele: FAM-ACACCAAAAAGCTTAACTGTA-MGB |
|  |  | R: AAGAGCCCTTTACCACACTCTCA | A allele: HEX-ACCAAAAAGCTTACTATAA-MGB |
| RT | miR-21 | CTCAACTGGTGTCGTGGAGTCGGCAATTCAGTTGAGTCAACATC |  |
| RT | *U6* | AAAATATGGAACGCTTCACG |  |
| RT-qPCR | *ALPP* | F: TCTGGGTACTCAGGGTCTGG | R: ATCGCTACGCAGCTCATCTC |
| RT-qPCR | *IGF2BP1* | F: GGCCATCGAGAATTGTTGCAG | R: CCAGGGATCAGGTGAGACTG |
| RT-qPCR | *ACTINB* | F: CATGTACGTTGCTATCCAGGC | R: CTCCTTAATGTCACGCACGAT |
| RT-qPCR | miR-21 | F: ACACTCCAGCTGGG TAGCTTATCAGACTGA | R: TGGTGTCGTGGAGTCG |
| RT-qPCR | *U6* | F: CGCTTCGGCAGCACATATACTAAAATTGGAAC | R: GCTTCACGAATTTGCGTGTCATCCTTGC |
| miR-21 mimic control | | Sense: UUCUCCGAACGUGUCACGUUU | Antisense: AAACGUGACACGUUCGGAGAA |
| miR-21 mimic | | Sense: UAGCUUAUCAGACUGAUGUUGA | Antisense: UAGCUUAUCAGACUGAUGUUGA |
| miR-21 inhibit control | | UUCUCCGAACGUGUCACGUUU |  |
| miR-21 inhibit | | UCAACAUCAGUCUGAUAAGCUA |  |

**Supplementary Table 2. Clinical characteristics of colorectal cancer tissues**

| Variables | N = 111 |
| --- | --- |
| Age (years, mean ± SD) | 61.6 ± 12.6 |
| Gender |  |
| Male | 64 |
| Female | 47 |
| Tumor site |  |
| Colon | 52 |
| Rectum | 59 |
| Histological grade |  |
| Low | 1 |
| Intermediate | 102 |
| High | 7 |
| Dukes stage |  |
| A | 3 |
| B | 63 |
| C | 44 |
| D | 1 |

**Supplementary Table 3. Demographic and clinical characteristics of colorectal cancer cases and controls**

| Variables | Cases (%), n = 1147 | Controls (%), n = 1203 | *P*^a^ |
| --- | --- | --- | --- |
| Age (mean ± SD) | 60.0 ± 12.6 | 59.9 ± 14.3 | 0.751 |
| Gender |  |  |  |
| Male | 702 (61.2) | 698 (58.0) | 0.116 |
| Female | 445 (38.8) | 505 (42.0) |  |
| Smoking status |  |  |  |
| Never | 736 (64.2) | 811 (67.4) | 0.097 |
| Ever | 411 (35.8) | 392 (32.6) |  |
| Drinking status |  |  |  |
| Never | 823 (71.8) | 898 (74.7) | 0.113 |
| Ever | 324 (28.2) | 305 (25.3) |  |
| Family history of cancers |  |  |  |
| No | 904 (78.8) | 1076 (89.4) | < 0.001 |
| Yes | 243 (21.2) | 127 (10.6) |  |
| Tumor site |  |  |  |
| Colon | 559 (48.7) |  |  |
| Rectum | 588 (51.3) |  |  |
| Histological grade |  |  |  |
| Low | 85 (7.4) |  |  |
| Intermediate | 880 (76.7) |  |  |
| High | 182 (15.9) |  |  |
| Dukes stage |  |  |  |
| A | 97 (8.4) |  |  |
| B | 494 (43.1) |  |  |
| C | 422 (36.8) |  |  |
| D | 134 (11.7) |  |  |

SD, standard deviation.

^a^Two-sided χ^2^ test for the frequency distributions of selected variables between the cases and controls.

**Supplementary Table 4. The baseline characteristics of the selected SNPs**

| SNPs | Genes | Location^a^ | Alleles^b^ | Cases^c^ (n = 1,147) | Controls^c^ (n = 1,203) | MAF^d^ (cases/controls) | *P*_HWE_^e^ |
| --- | --- | --- | --- | --- | --- | --- | --- |
| rs2273847 | *GALNT12* | Chr9: 101611545 | A/G | 531/485/131 | 584/507/112 | 0.326/0.304 | 0.897 |
| rs6504593 | *IGF2BP1* | Chr17: 47132819 | T/C | 646/422/79 | 735/405/63 | 0.253/0.221 | 0.460 |
| rs1049109 | *ALPP* | Chr2: 233247027 | C/T | 654/431/62 | 758/393/52 | 0.242/0.207 | 0.906 |
| rs6108 | *SERPINA5* | Chr14: 95058631 | A/T | 777/335/35 | 786/362/55 | 0.177/0.196 | 0.112 |
| rs7337488 | *SETDB2* | Chr13: 50068628 | G/A | 892/254/1 | 963/239/1 | 0.112/0.105 | <0.001 |

OR, odds ratio; CI, confidence interval.

^a^Location in GRCh 37.

^b^Major/minor alleles.

^c^Numbers of major homozygote/heterozygote/minor homozygote.

^d^Minor allele frequency.

^e^HWE, Hardy-Weinberg equilibrium in the controls.

**Supplementary Table 5****. Stratification analysis by demographic characteristics for rs6504593 and colorectal cancer risk**

| Variables | Genotypes (cases/controls) | | | | OR (95% CI)^a^ | *P*^a^ |
| --- | --- | --- | --- | --- | --- | --- |
|  | TT |  | TC/CC | |  |  |
|  | N | % | N | % |  |  |
| Age (years) | |  |  | |  |  |
| ≤ 60 | 318/339 | 55.5/61.1 | 255/216 | 44.5/38.9 | 1.26 (0.99-1.60) | 0.063 |
| > 60 | 328/396 | 57.1/61.1 | 246/252 | 42.9/38.9 | 1.20 (0.95-1.51) | 0.136 |
| Gender |  |  |  | |  |  |
| Male | 399/432 | 56.8/61.9 | 303/266 | 43.2/38.1 | 1.23 (0.99-1.53) | 0.062 |
| Female | 247/303 | 55.5/60.0 | 198/202 | 44.5/40.0 | 1.16 (0.89-1.51) | 0.274 |
| Smoking status | |  |  | |  |  |
| Never | 408/501 | 55.4/61.8 | 328/310 | 44.6/38.2 | **1.30 (1.07-1.60)** | 0.010 |
| Ever | 238/234 | 57.9/59.7 | 173/158 | 42.1/40.3 | 1.07 (0.80-1.43) | 0.640 |
| Drinking status | |  |  | |  |  |
| Never | 465/560 | 56.5/62.4 | 358/338 | 43.5/37.6 | **1.28 (1.05-1.55)** | 0.013 |
| Ever | 181/175 | 55.9/57.4 | 143/130 | 44.1/42.6 | 1.12 (0.80-1.55) | 0.512 |
| Family history of cancers | | |  |  |  |  |
| No | 516/648 | 57.1/60.2 | 388/428 | 42.9/39.8 | 1.14 (0.95-1.36) | 0.163 |
| Yes | 130/87 | 53.5/68.5 | 113/40 | 46.5/31.5 | **2.03 (1.28-3.23)** | 0.003 |

OR, odds ratio; CI, confidence interval.

^a^Adjusted for age, sex, smoking and drinking status in logistic regression model.

**Supplementary Table 6. Stratification analysis by demographic characteristics for rs1049109 and colorectal cancer risk**

| Variables | Genotypes (cases/controls) | | | | OR (95% CI)^a^ | *P*^a^ |
| --- | --- | --- | --- | --- | --- | --- |
|  | CC |  | CT/TT | |  |  |
|  | N | % | N | % |  |  |
| Age (years) | |  |  | |  |  |
| ≤ 60 | 342/349 | 59.7/62.9 | 231/206 | 40.3/37.1 | 1.18 (0.92-1.51) | 0.184 |
| > 60 | 312/409 | 54.4/63.1 | 262/239 | 45.6/36.9 | **1.47 (1.16-1.85)** | 0.001 |
| Gender |  |  |  | |  |  |
| Male | 402/435 | 57.3/62.3 | 300/263 | 42.7/37.7 | 1.23 (0.99-1.52) | 0.067 |
| Female | 252/323 | 56.6/64.0 | 193/182 | 43.4/36.0 | **1.42 (1.08-1.85)** | 0.011 |
| Smoking status | |  |  | |  |  |
| Never | 419/510 | 56.9/62.9 | 317/301 | 43.1/37.1 | **1.30 (1.06-1.60)** | 0.012 |
| Ever | 235/248 | 57.2/63.3 | 176/144 | 42.8/36.7 | 1.27 (0.95-1.70) | 0.101 |
| Drinking status | |  |  | |  |  |
| Never | 466/573 | 56.6/63.8 | 357/325 | 43.4/36.2 | **1.37 (1.13-1.66)** | 0.002 |
| Ever | 188/185 | 58.0/60.7 | 136/120 | 42.0/39.3 | 1.14 (0.82-1.59) | 0.432 |
| Family history of cancers | | |  |  |  |  |
| No | 524/674 | 58.0/62.6 | 380/402 | 42.0/37.4 | **1.21 (1.01-1.45)** | 0.037 |
| Yes | 130/84 | 53.5/66.1 | 113/43 | 46.5/33.9 | **1.69 (1.06-2.62)** | 0.026 |

OR, odds ratio; CI, confidence interval.

^a^Adjusted for age, sex, smoking and drinking status in logistic regression model.

**Supplementary Table 7. Associations between rs1049109 genotype and clinical characteristics of colorectal cancer**

| Variables | Genotypes | | OR (95% CI)^a^ | *P*^a^ |
| --- | --- | --- | --- | --- |
|  | CC, n (%) | CT/TT, n (%) |  |  |
| Controls (n = 1203) | 758 (63.0) | 445 (37.0) | 1.00 |  |
| Cases (n = 1147) | 654 (57.0) | 493 (43.0) | **1.28 (1.09-1.51)** | 0.003 |
| Tumor site |  |  |  |  |
| Colon | 327 (58.5) | 232 (41.5) | **1.25 (1.02-1.53)** | 0.034 |
| Rectal | 327 (55.6) | 261 (44.4) | 1.19 (0.97-1.45) | 0.092 |
| Histological grade |  |  |  |  |
| Low | 45 (52.9) | 40 (47.1) | 1.52 (0.98-2.37) | 0.062 |
| Intermediate | 503 (57.2) | 377 (42.8) | **1.27 (1.06-1.52)** | 0.009 |
| High | 106 (58.2) | 76 (41.8) | 1.23 (0.89-1.68) | 0.212 |
| Dukes stage |  |  |  |  |
| A + B | 326 (55.2) | 265 (44.8) | **1.38 (1.13-1.68)** | 0.002 |
| C + D | 328 (59.0) | 228 (41.0) | 1.18 (0.96-1.45) | 0.114 |

OR, odds ratio; CI, confidence interval.

^a^Adjusted for age, sex, smoking and drinking status in logistic regression model.

**Supplementary figure legends**

**Supplementary Figure 1.** The correlation of miR-21 and *IGF2BP1* expression in colorectal cancer tissues (A) and normal colorectal tissues (B). Association of the expression of miR-21 and *IGF2BP1* in TCGA data (C).

**Supplementary Figure 2.** Relationship between rs6504593 genotypes and relative *IGF2BP1* expression in TCGA data of (A) colorectal cancer tissues and (B) normal colorectal tissues**.** The *IGF2BP1* mRNA expression levels were log2 transformed. The *P* value was calculated using ANOVA.

**Supplementary Figure 3.** Transient transfection with miR-21 mimics or inhibitor regulated HCT116 cell proliferation and apoptosis. (A) Cell proliferation activity was measured by the CCK8 assay. *, *P* < 0.05. (B) The level of apoptosis was detected by flow cytometry. All bars represent the mean values ± SD. All results are representative of triplicate experiments. The *P* value was calculated by two-sided *t*-test. LR, early apoptotic cells; UR, terminal apoptotic cells.

**Supplementary Figure 4.** Transient transfection with miR-21 mimics regulated cell cycle in HCT116 (A) and SW620 (B) cells. The cell cycle progression was analyzed by flow cytometry. The results are the mean of three independent experiments ± SD, and the *P* value was calculated by two-sided *t*-test.

**Supplementary Figure 1:**


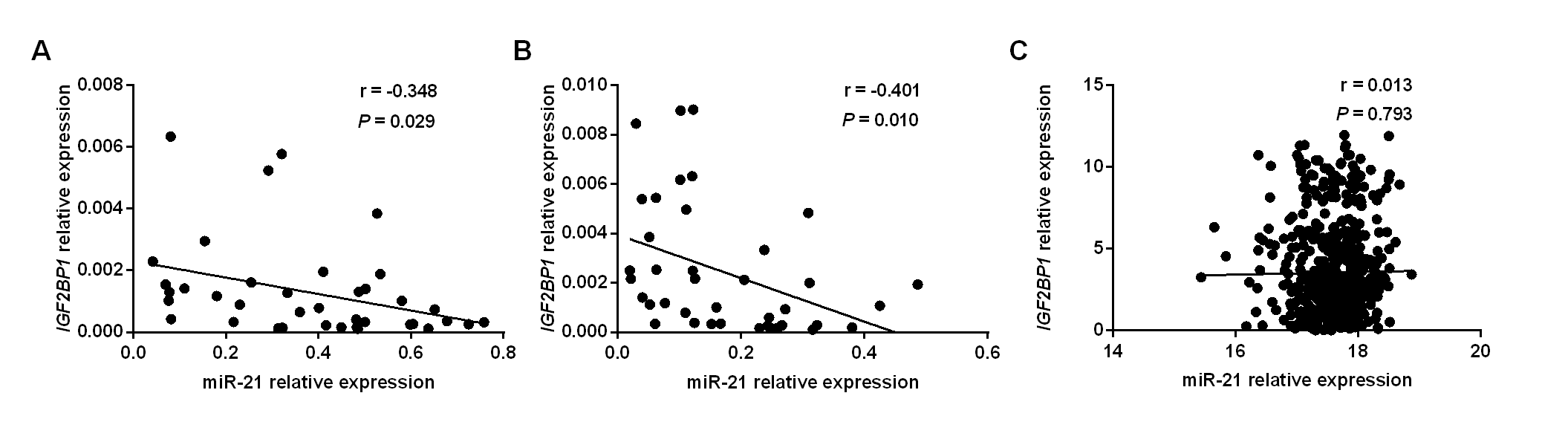


**Supplementary Figure 2:**


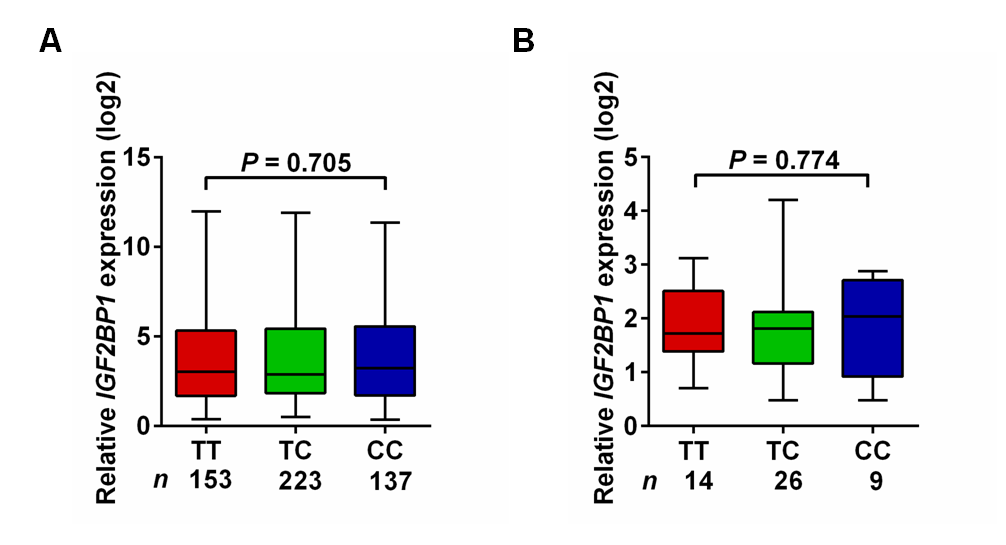


**Supplementary Figure 3:**


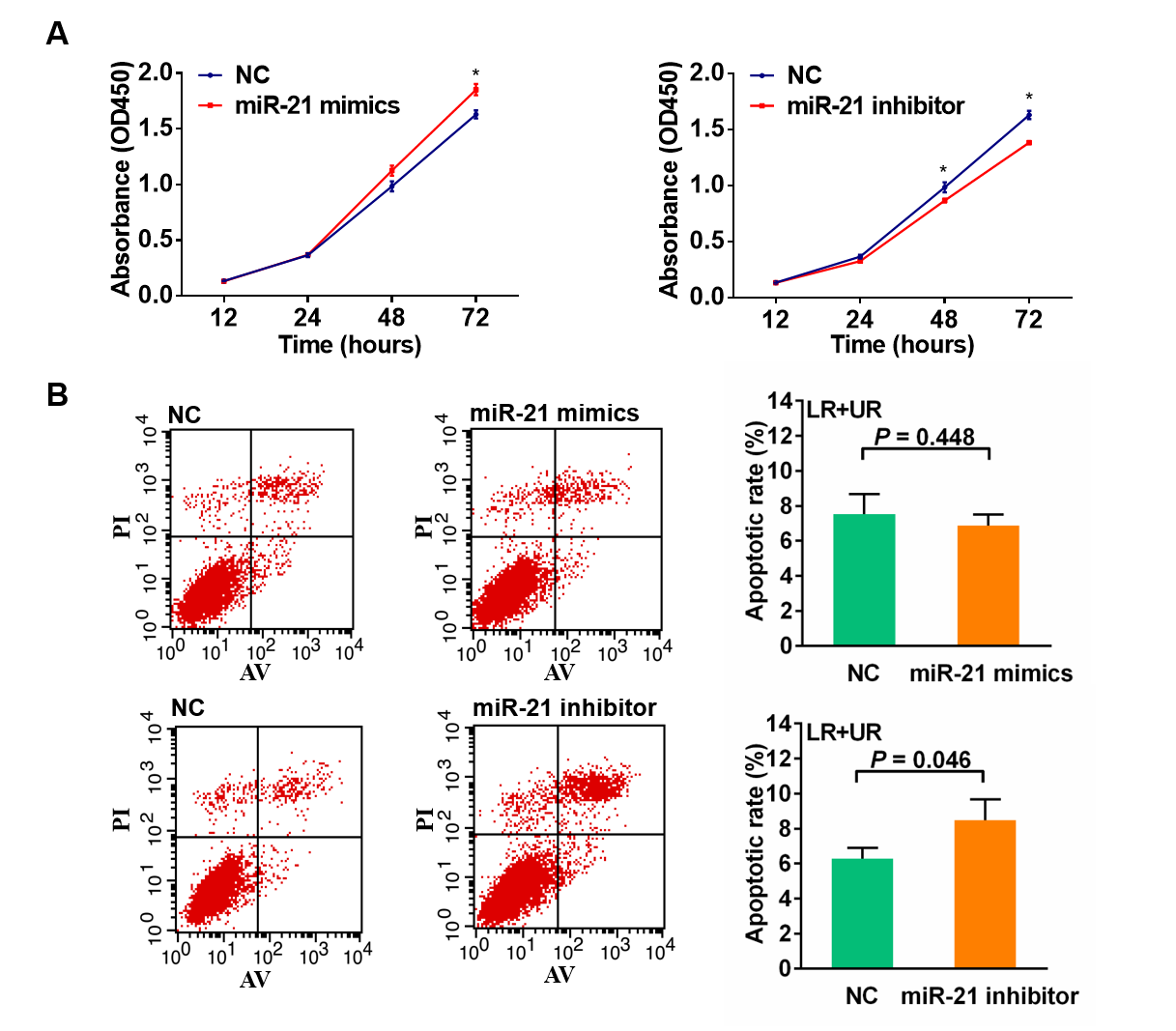


**Supplementary Figure 4:**


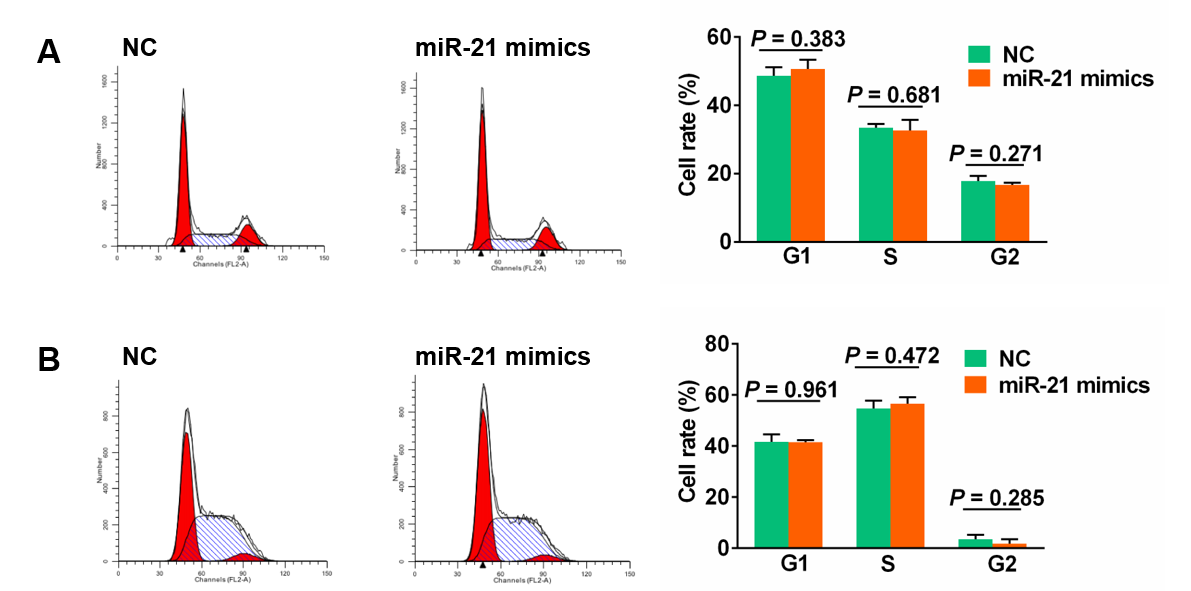

Supplement: Supplementary file 1 [file JCMM-23-2012-s001.docx]
